# 11β-Hydroxysteroid Dehydrogenase Type 1 Facilitates Osteoporosis by Turning on Osteoclastogenesis through Hippo Signaling

**DOI:** 10.7150/ijbs.82933

**Published:** 2023-07-15

**Authors:** Hanwen Li, Sihan Hu, Runze Wu, Hongyou Zhou, Kai Zhang, Ke Li, Wenzheng Lin, Qin Shi, Hao Chen, Shan Lv

**Affiliations:** 1 Department of Geriatric Endocrinology, Jiangsu Province Hospital and Nanjing Medical University First Affiliated Hospital, Nanjing 210029, Jiangsu, China.; 2 Department of Orthopedics, Affiliated Hospital of Yangzhou University, Yangzhou, China.; 3 Department of Orthopedic, First Affiliated Hospital of Soochow University, Suzhou, China.; 4 Department of Endocrinology, Changshu No.2 People's Hospital, Changshu 215500, Jiangsu province, China.; 5 Institute of Translational Medicine, Medical College, Yangzhou University, Yangzhou, China.; 6 Orthopedic Institute of Soochow University, Suzhou, China.

**Keywords:** 11β-hydroxysteroid dehydrogenase type 1, endogenous glucocorticoid, osteoclast, H type vessel, Hippo signaling

## Abstract

11β-hydroxysteroid dehydrogenase type 1 (11β-HSD1) is a key enzyme that transform cortisone to cortisol, which activates the endogenous glucocorticoid function. 11β-HSD1 has been observed to regulate skeletal metabolism, specifically within osteoblasts. However, the function of 11β-HSD1 in osteoclasts has not been elucidated. In this study, we observed increased 11β-HSD1 expression in osteoclasts within an osteoporotic mice model (ovariectomized mice). Then, 11β-HSD1 global knock-out or knock-in mice were employed to demonstrate its function in manipulating bone metabolism, showing significant bone volume decrease in 11β-HSD1 knock-in mice. Furthermore, specifically knock out 11β-HSD1 in osteoclasts, by crossing cathepsin-cre mice with 11β-HSD1^flox/flox^ mice, presented significant protecting effect of skeleton when they underwent ovariectomy surgery. *In vitro* experiments showed the endogenous high expression of 11β-HSD1 lead to osteoclast formation and maturation. Meanwhile, we found 11β-HSD1 facilitated mature osteoclasts formation inhibited bone formation coupled H type vessel (CD31^hi^Emcn^hi^) growth through reduction of PDFG-BB secretion. Finally, transcriptome sequencing of 11β-HSD1 knock in osteoclast progenitor cells indicated the Hippo pathway1 was mostly enriched. Then, by suppression of YAP expression in Hippo signaling, we observed the redundant of osteoclasts formation even in 11β-HSD1 high expression conditions. In conclusion, our study demonstrated the role of 11β-HSD1 in facilitating osteoclasts formation and maturation through the Hippo signaling, which is a new therapeutic target to manage osteoporosis.

## Introduction

Osteoporosis has become a global health issue in recent years. It is characterized by the loss of bone mass and strength which leads to an increased risk of fracture, contributing to morbidity and mortality^1^, ^2^. Bone is a unique connective tissue which constantly undergoes remodeling, which involves bone formation by osteoblasts and bone resorption by osteoclasts^3^. Estrogen deficiency is one of the main causes leads to the pathogenesis of osteoporosis, which is widely observed in the post-menopausal population. A dramatic chaos in estrogen level can change the status of endogenous hormones and disrupt the bone formation-resorption balance. On the other hand, in the elderly, age-associated hormonal imbalance is involved in the pathogenesis of various diseases, including osteoporosis^4^, which might be associated with increased endogenous inflammation environment, like TNF-α^5^ and IL-17a^6^ that triggers endogenous hormone changes to induce bone loss.

Postmenopausal osteoporosis remains the most common type of osteoporosis. The main pathological change is estrogen deficiency. Studies have confirmed that the activity of bone cells is drastically altered due to estrogen deficiency^7^. Many studies have attempted to understand phenomenon of reduced bone mineral density due to estrogen deficiency. Studies have shown that, when estrogen binds to preosteoclasts, osteoclast formation and differentiation are reduced, while osteoclast apoptosis is increased^8^. At the same time, estrogen can promote the osteogenic differentiation of mesenchymal stem cells (MSCs) and the maturation of osteoblasts, thereby promoting bone formation. Emerton et al. demonstrated that estrogen deficiency leads to increased apoptosis in osteocytes^9^. Wu et al.^10^ suggested that estrogen deficiency causes senescence of bone marrow cells in OVX mice. They observed upregulation of JAK2/STAT3 along with SASP factors in microenvironment of osteoporosis, which can be reversed by estrogen. In addition, estrogen itself interacts with various immune cells and also affects bone mineral density^11^, ^12^.

Studies have confirmed that helper cells in bone tissue are essential for bone homeostasis. For example, H type vessel (Emcn^hi^CD31^hi^) was found in the bone marrow cavity. It is thought to promote osteogenesis^13^, ^14^ and Emcn^hi^CD31^hi^ endothelial levels can be regulated by PDGF-BB secreted by pre-osteoclasts, providing a theoretical link between osteoclasts and H type vessel^15^. As observed, osteoporosis is one of the side effects of glucocorticoid (GC) therapy. Especially after SARS-CoV-2, a large population of people suffered from osteoporosis and osteonecrosis after taking higher or longer doses of corticosteroids^16^. However, these corticosteroids were taken from outside our body. It has been widely studied the role of exogenous glucocorticoids in osteoporosis, but how do endogenous glucocorticoids affect bone is still a confusion. 11β-Hydroxysteroid dehydrogenase (11β-HSD) is an intracellular enzyme^17^, converts the hormonally inactive glucocorticoid cortisone to become the active one, including 11β-HSD1 and 11β-HSD2. Recent researchers observed that 11β-HSD1 is closely associated with osteoporosis^18^. In human bone tissue, 11β-HSD1 expression and activity increased with aging, suggesting that 11β-HSD1 may be intrinsically related to osteoporosis^19^. In a clinical case of a 6.9 years old boy with defect of 11β-HSD1, who presented accelerated bone age (11.5 years)^20^. 11β-HSD1 in osteoblasts had been shown to be associated with inflammation, aging, and hormone-induced osteoporosis^21^-^23^. Selective inhibitors of 11β-HSD1 had been shown as a treatment for osteoporosis. Researchers found that treating ovariectomized rats with Piper Sarmentosum, an inhibitor of 11β-HSD1, improved the fracture callus score in the ovariectomized rats^24^. Zhou et al.^25^ observed that 11β-HSD2 also plays a role in regulation of bone homeostasis by manipulating endogenous glucocorticoids. However, all these researchers agree on that, no matter 11β-HSD1 or 11β-HSD2 is exerting their function in osteoporosis by affecting osteoblast lineage cells^26^, but no one had explored the function of 11β-HSD in osteoclasts.

In current study, we further explored the position of 11β-HSD1 in osteoclast differentiation, in order to better understand the endogenous hormone regulation in bone remodeling. We found 11β-HSD1 is indeed involved in osteoclasts formation, which also influences the function of bone coupling vessel type H, which makes a supplementation of the 11β-HSD1 function in osteoporosis. The undermined mechanism might be through the Hippo signaling pathway. Moreover, we demonstrated further that 11β-HSD1 knock out or inhibition in osteoclasts can effectively reverse osteoporosis in estrogen deficiency mice, which could be a potential therapeutic target for endogenous corticosteroids induced osteoporosis.

## Results

### Increased 11β-HSD1 expression and osteoclast activation in ovariectomy (OVX) mice

**Figure 1:**
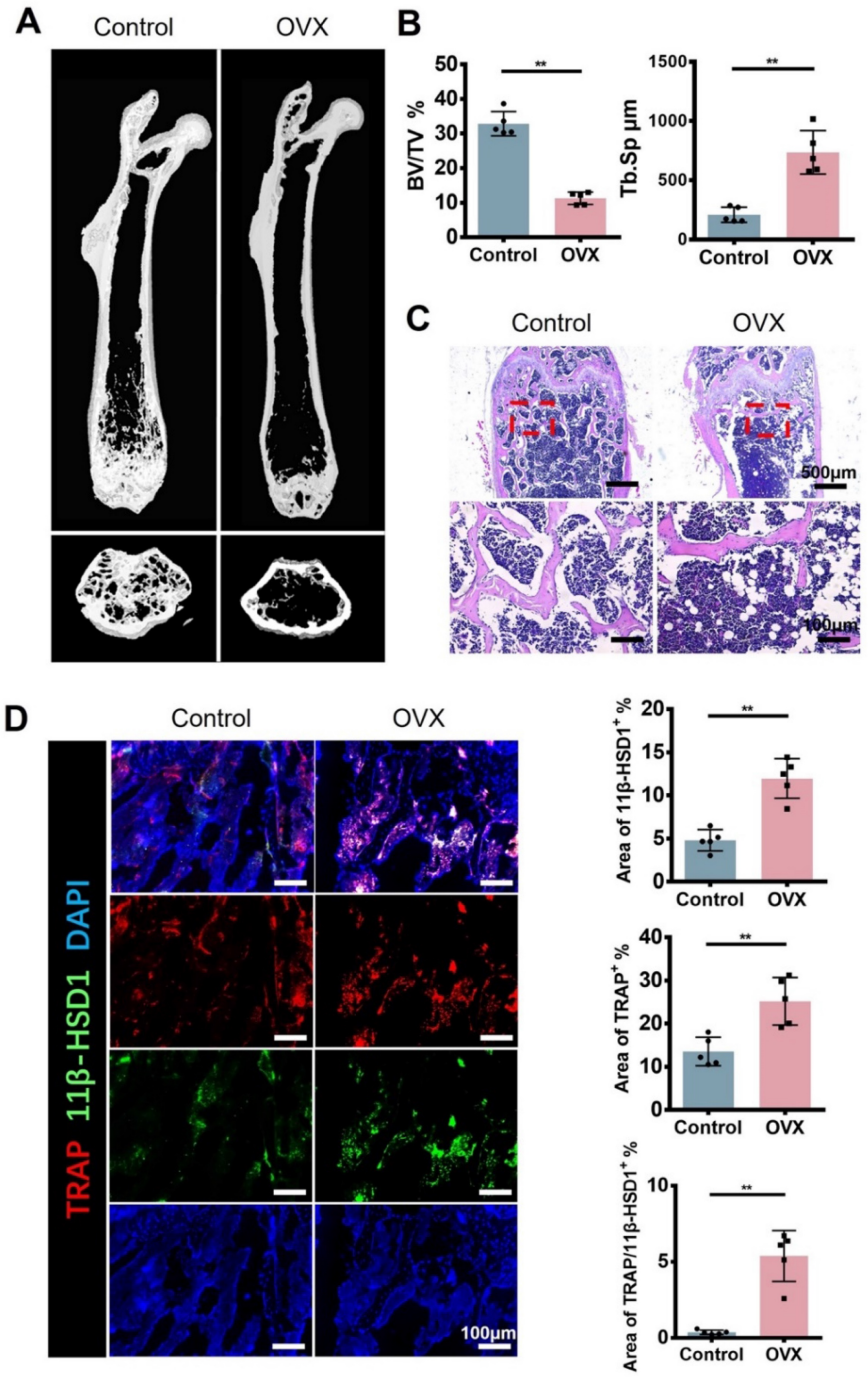
** 11β-HSD1 expression and osteoclast number increased in ovariectomized (OVX) mice. A.** Representative μCT images of 8w-OVX mice femur and the control. **B.** Quantitative μCT analysis of trabecular bone volume to tissue volume (BV/TV) and trabecular separation (Tb. Sp) of femora. n = 5 per group. **C.** Representative H&E staining images of 8w-OVX mice femur and the control. **D.** Representative IF images of 11β-HSD1 (green) and TRAP (red) staining in 8w-OVX mice femur and the control (Left). Quantification of 11β-HSD1 and TRAP staining (Right). (*means *P* value<0.05, **means *P* value<0.01)

In order to figure out the expression of 11β-HSD1 in osteoporosis development, we constructed ovariectomy (OVX) model to mimic osteoporosis. The Micro-CT (μCT) analysis of OVX mice femur showed significant decreased bone volume (Figure 1A and B), which is in accordance with previous studies. Analysis of μCT demonstrated that trabecular bone volume to tissue volume (BV/TV) was lowered in the OVX group and trabecular separation/spacing (Tb. Sp) was increased in the OVX group. Similarly, H&E staining of mice femurs revealed the decrease of bone trabeculae in the OVX mice as observed in μCT (Figure 1C). Then, immunofluorescent (IF) staining was performed to evaluate the expression of TRAP and 11β-HSD1. The results showed significant increase of TRAP positive cells and 11β-HSD1 positive cells at the same time, most of which were colocalized (Figure 1D). These data clearly show the increased expression of TRAP and 11β-HSD1 in OVX mice is accompanied with decreased bone volume.

### High expression of 11β-HSD1 induced osteoporosis *in vivo*

**Figure 2:**
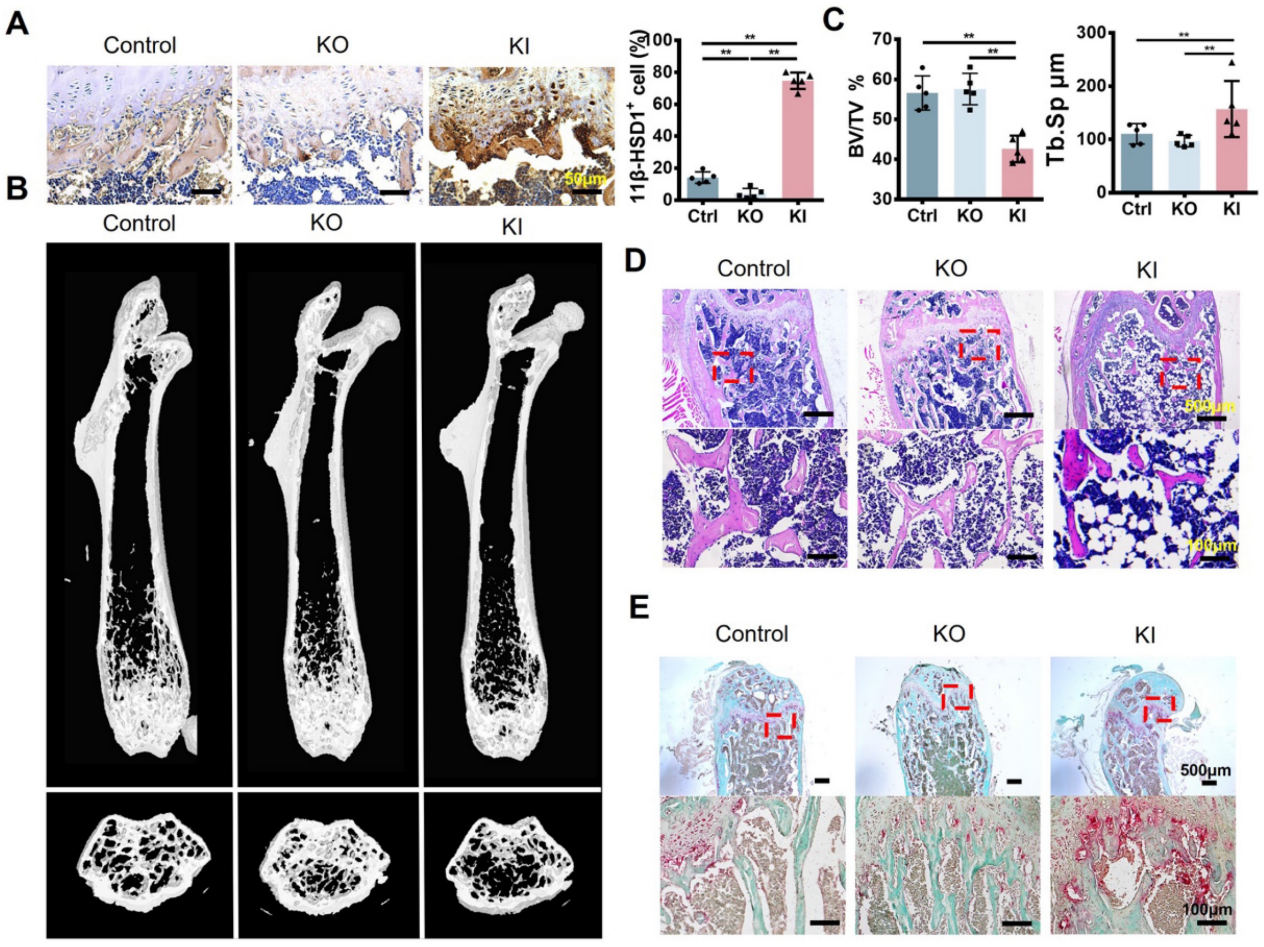
** High expression of 11β-HSD1 induced osteoporosis *in vivo.* A.** Representative immumohistochemical images of 11β-HSD1 staining in the control, 8w-KO and 8w-KI mice femur (Left). Quantification of 11β-HSD1 staining (Right). **B.** Representative μCT images of mice femurs from the control, 8w-KO and 8w-KI groups. **C.** Quantitative μCT analysis of trabecular bone volume to tissue volume (BV/TV) and trabecular separation (Tb. Sp). n = 5 per group. **D.** Representative H&E staining images of mice femurs from the control, 8w-KO and 8w-KI groups. **E.** Representative TRAP staining of mice femurs from the control, 8w-KO and 8w-KI groups. (*means *P* value<0.05, **means *P* value<0.01)

To verify if 11β-HSD1 contributes to osteoporosis, we constructed two different models with 11β-HSD1 knock out (KO) and knock in (KI). 11β-HSD1 staining confirmed that 11β-HSD1 expression increased in KI group while opposite in the other (Figure 2A). As expected, μCT analysis showed the bone volume in KI group was significantly decreased, while the KO group of mice did not show bone volume change (Figure 2B and C). Similarly, H&E staining revealed the trabecular bone loss in the KI group (Figure 2D). Moreover, we also performed TRAP staining to see how osteoclast number changes along with 11β-HSD1 expression. The data showed osteoclast number in the KI group increased dramatically as compared to the control and KO groups (Figure 2E). The results above indicate that 11β-HSD1 overexpression induces osteoporosis, which is similar like that in the OVX mice.

### 11β-HSD1 promotes osteoclastogenesis *in vitro*

**Figure 3:**
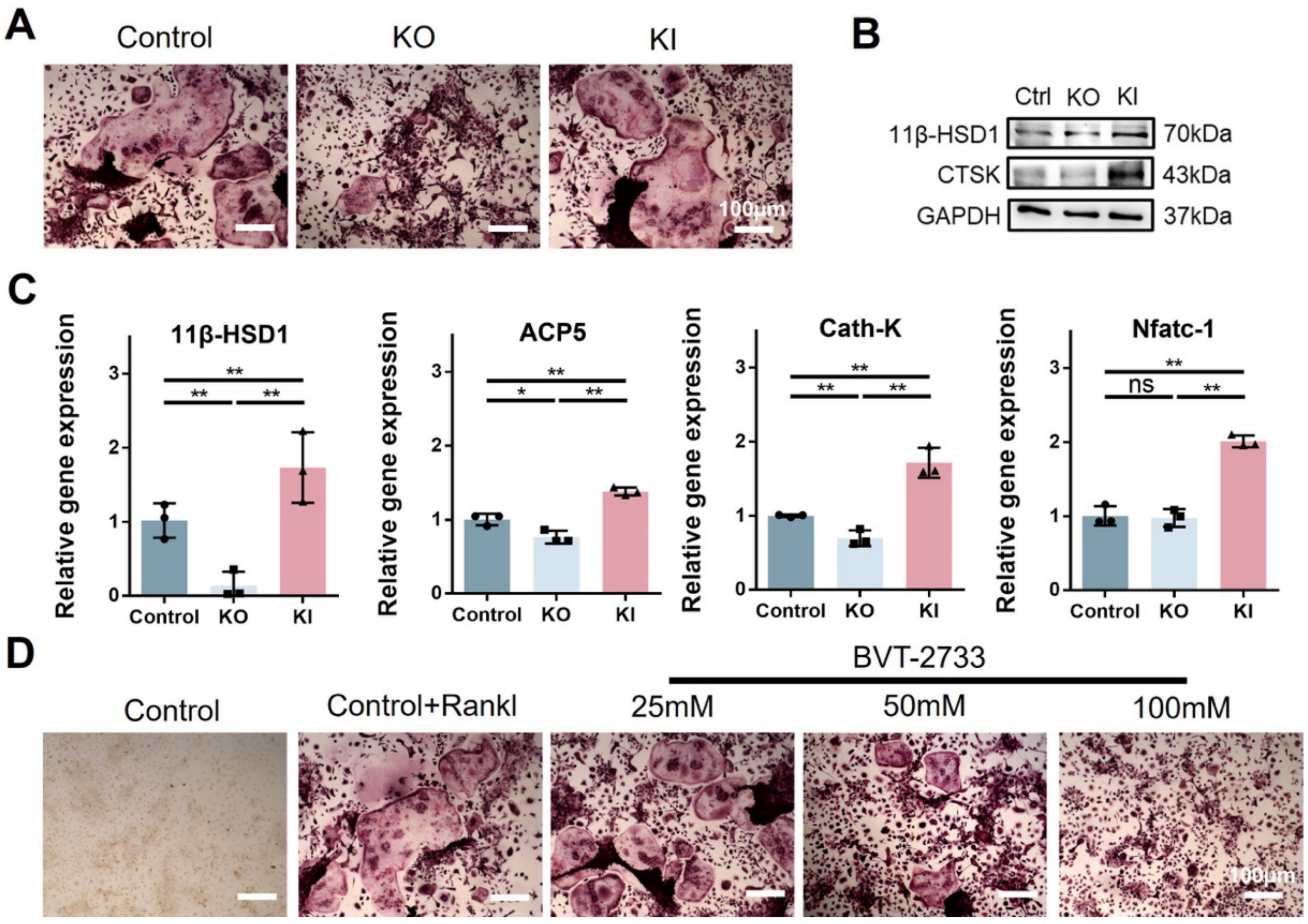
** 11β-HSD1 promotes osteoclastogenesis *in vitro*. A.** BMMs were isolated from the wild type, KO and KI groups. After induction with osteoclastic differentiation medium for 7 days, TRAP staining was performed to evaluate osteoclast formation.** B.** Western blot (WB) evaluation of 11β-HSD1 and CTSK expression change in BMMs from the wild type, KO and KI groups.** C.** RT-qPCR analysis of 11β-HSD1, ACP5, Cath-K and Nfatc-1.** D.** BVT-2733 with different concentrations were added into osteoclast incubation medium. Trap staining was performed to evaluate the osteoclast formation. (*means *P* value<0.05, **means *P* value<0.01)

Since osteoclast activation is common in osteoporosis, and we have observed the high expression of 11β-HSD1 in OVX mice femurs. Hence, to further investigate the regulation of 11β-HSD1 in osteoclast formation or activity, we performed* in vitro* study with bone marrow monocytes from KO and KI mice separately. BMMs extracted from KI, KO and wild type mice underwent osteoclast differentiation with M-CSF and RANKL. Osteoclast formation was evaluated by TRAP staining, showing increased osteoclast formation in the KI group as compared with the control one, while it was significantly reduced in the KO group (Figure 3A). We confirmed the expression of 11β-HSD1 and osteoclast maker CTSK with Western Blot (WB), showing the CTSK expression was suppressed in the BMMs from the KO mice, but increased in the BMMs from the KI mice (Figure 3B). The same trend was also observed with RT-qPCR analysis of osteoclast-related genes expression (Figure 3C), which also confirmed that 11β-HSD1 was successfully reduced in BMMs from the KO mice and increased in BMMs from the KI mice. Finally, we used BVT-2733, a selective 11β-HSD1 inhibitor, to artificially inhibit the 11β-HSD1 expression. After osteoclastic induction, TRAP staining showed that 11β-HSD1 inhibition significantly suppressed osteoclast formation *in vitro* in a BVT2733 concentration-dependent manner (Figure 3D). The results above fully demonstrate the key role of 11β-HSD1 in osteoclastogenesis.

### Conditional knock out 11β-HSD1 in osteoclast rescued osteoporosis *in vivo*

**Figure 4:**
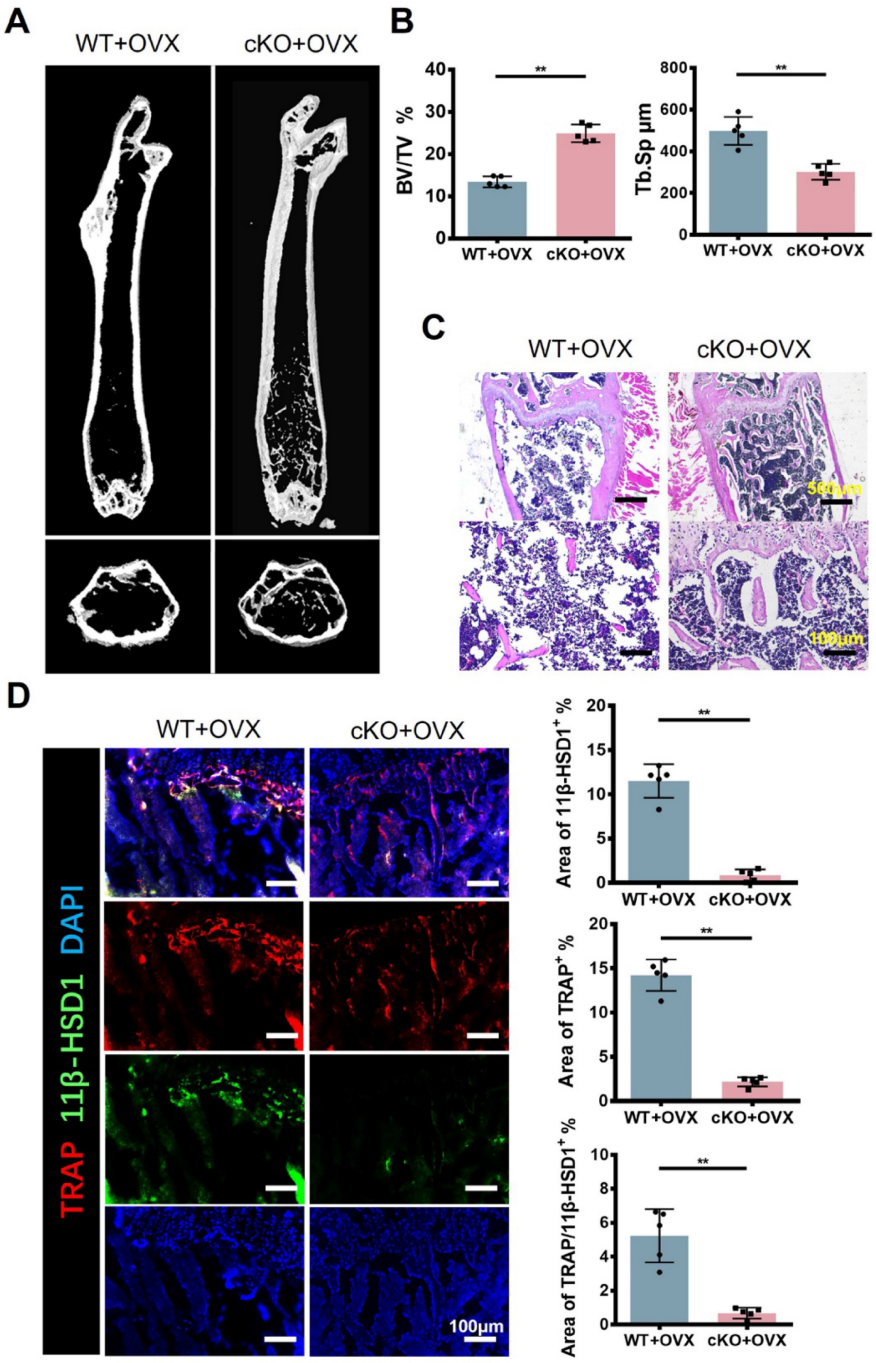
** Conditional knock out 11β-HSD1 in osteoclast rescued osteoporosis *in vivo*. A.** Representative μCT images of the femurs from 8w-cKO or wild type mice in OVX conditions. **B.** Quantitative μCT analysis of BV/TV and Tb. Sp. n = 5 per group.** C.** Representative H&E staining images of the femurs. **D.** Representative IF images showing 11β-HSD1 (green) and TRAP (red) staining in the femurs from cKO or wild type mice in OVX conditions (Top). Quantification of 11β-HSD1 and TRAP staining (Bottom). (*means *P* value<0.05, **means *P* value<0.01)

Referring to previous studies^27^, bone marrow cells from KI, KO and WT mice were used to detect osteogenesis-related genes. RT-qPCR results showed that 11β-HSD1 inhibited osteoblast proliferation (Figure S2). To further investigate the function of 11β-HSD1 in osteoclasts, we constructed 11β-HSD1 conditional knock out mice by crossing Ctsk-Cre mice with 11β-HSD1^flox/flox^ mice (cKO), since Ctsk was considered as a recognized osteoclast marker. Then, we performed OVX model in these mice to induce osteoporosis *in vivo*. Micro-CT analysis showed the bone volume in cKO mice was higher than that in control group under OVX conditions (Figure 4A and B). By H&E staining, we observed more and thicker bone trabeculae in 11β-HSD1 knockout mice with OVX surgery as compared to its control (Figure 4C). Then, IF staining showed that after conditional deletion of 11β-HSD1 in osteoclasts, the osteoclast number was decreased as well, along with the decrease of 11β-HSD1 expression (Figure 4D).

### 11β-HSD1 suppressed H type vessel formation

**Figure 5:**
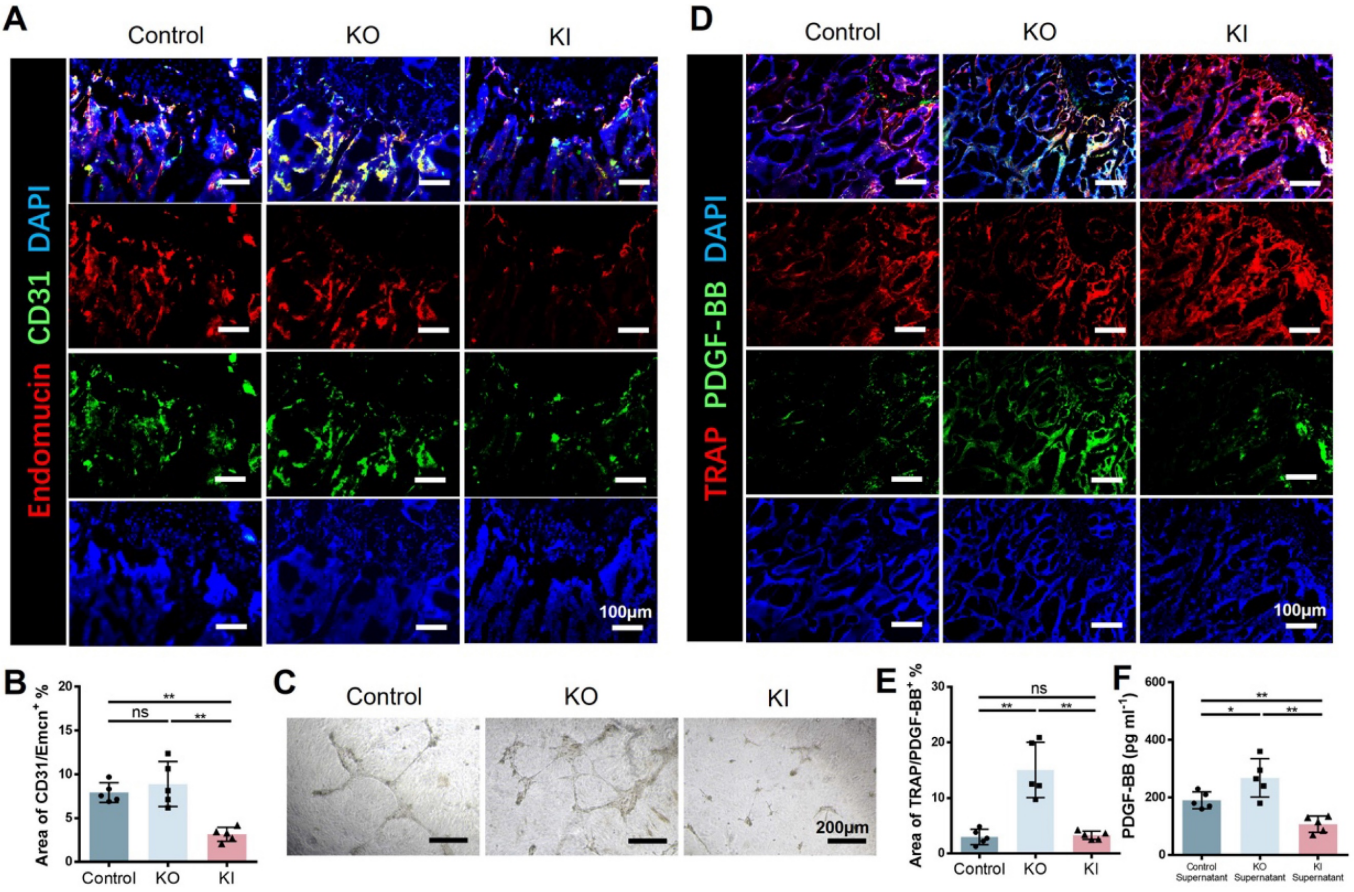
** 11β-HSD1 suppressed H type vessel formation. A.** Representative IF images of Endomucin (Emcn, red), CD31 (green) and DAPI (blue). **B.** Quantitative analysis of the area of H type vessel (CD31^hi^Emcn^hi^, yellow) near femoral growth plate in control, KO and KI groups. **C.** Representative images of tube formation assay with the supernatant of osteoclast induction medium from the control, KO and KI groups. **D.** Representative IF images of TRAP (red), PDGF-BB (green) and DAPI (blue). **E.** Quantitative analysis of the area of TRAP with PDGF-BB. **F.** PDGF-BB Elisa analysis of the supernatant in osteoclast induction from the control, KO and KO group.

Skeletal growth is closely associated with vessels. H type vessel has recently been verified to be a subtype vessel (CD31^hi^Emcn^hi^) that coupling with osteogenesis^15^. Xie et al reported that pre-osteoclast secreted platelet-derived growth factor-BB (PDGF-BB) is a major source to induce H type vessel formation. Therefore, we speculate that 11β-HSD1 promoted osteoclast formation and maturation may reduce the pre-osteoclast number, which finally lead to decreased PDGF-BB secretion. To test this, we first performed coimmunostaining of CD31 and Emcn in the femurs of wild type (control), KO and KI mice, demonstrating that the area of H type vessel in the control and KO groups was significantly higher relative to the KI group (Figure 5A and B). Then we further detected the angiogenesis capacity with the pre-osteoclast supernatant from each group during osteoclasts induction. The supernatant of control and KO groups promoted vessel formation efficiently, while it can be rarely observed of the vessel formation in the supernatant of KI group (Figure 5C). Moreover, we detected the expression of PDGF-BB in the three groups of mice by co-staining of PDGF-BB and TRAP. Consistently, we observed the lowest PDGF-BB expression in the KI group within the TRAP positive cells (Figure 5D and E). Meanwhile, the PDGF-BB within the supernatant in each group was detected by Elisa analysis, revealing that the PDGF-BB content was significantly reduced in the KI group, which is in accordance with the *in vivo* data (Figure 5F). All these results above confirm that 11β-HSD1 suppresses H type vessel formation by promoting osteoclasts maturation, which further destructs osteogenic microenvironment.

### 11β-HSD1 promotes osteoclastogenesis through Hippo signaling pathway

**Figure 6:**
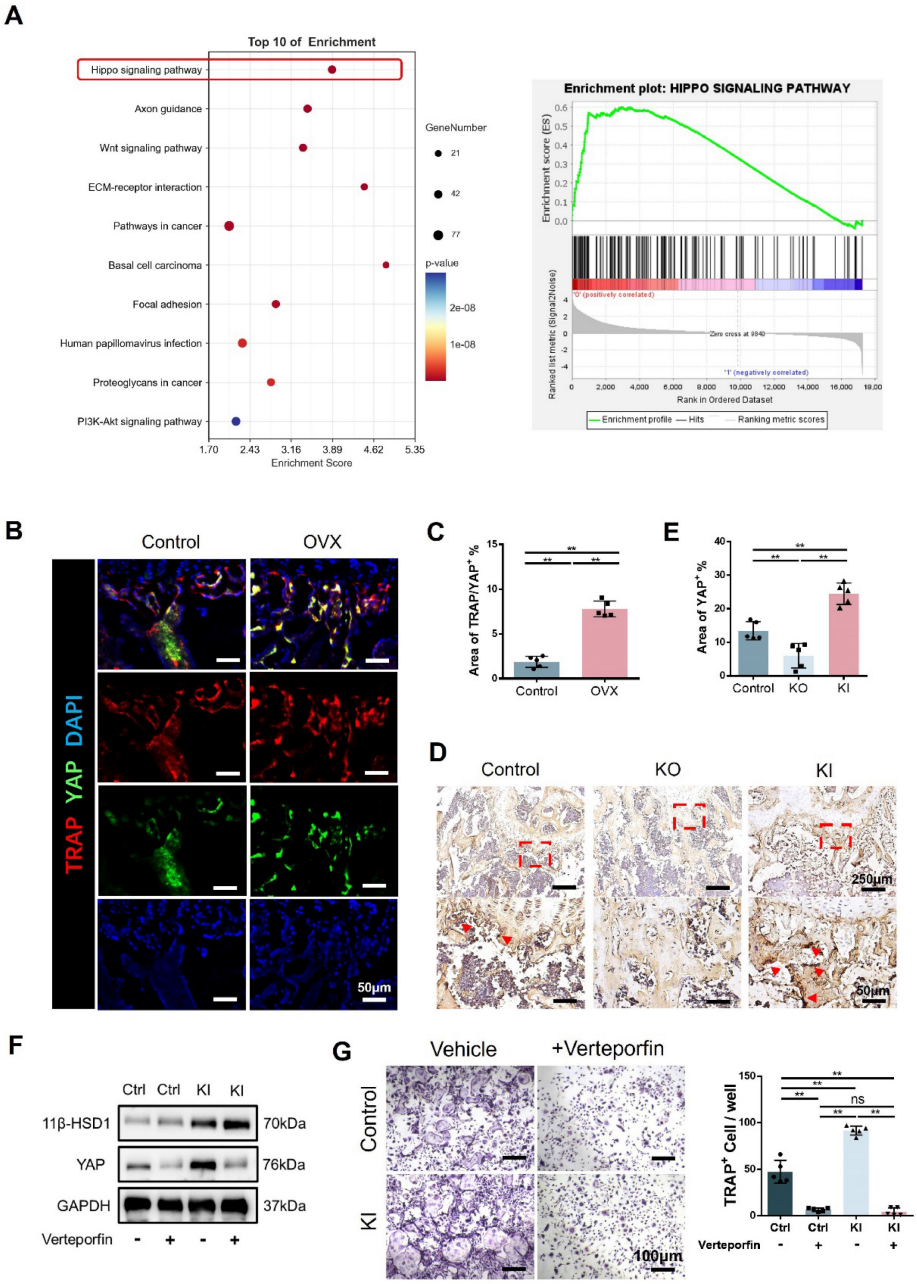
** 11β-HSD1 promotes osteoclastogenesis through Hippo signaling pathway. A.** Transcriptome analysis of the BMMs isolated from the control and KI femurs. **B.** Representative IF images of TRAP (red), YAP (green) and DAPI (blue). **C.** Quantitative analysis of the area of TRAP colocalized with YAP (yellow) near femoral growth plate. **D.** Representative immumohistochemical images showing YAP in the femors from control, KO and KI groups. **E.** Quantification analysis of YAP staining. **F.** WB evaluation of 11β-HSD1 and YAP expression change in BMMs under verteporfin conditions. **G.** Verteporfin was added into osteoclast incubation medium. TRAP staining was performed to evaluate the osteoclast formation (Left) and the quantitative analysis (Right).

To further study the relationship between 11β-HSD1 and osteoclasts, we analyzed the transcriptome of BMMs in control and KI groups. Transcriptome analysis showed that Hippo signaling is significantly enriched, indicating the key function of Hippo signaling in 11β-HSD1 regulated osteoclastogenesis (Figure 6A). As YAP is the key protein in the Hippo signaling pathway, we firstly evaluated the expression of YAP in osteoclast *in vivo*. We performed immunofluorescence staining in femurs from OVX mice. Coimmunostaining of TRAP and YAP revealed that the expression of YAP in TRAP cells was significantly increased with osteoclast activation (Figure 6B and C). Furthermore, immunohistochemical staining of YAP in the control, KO and KI groups also revealed the YAP expression increased in the KI group (Figure 6D and E). Next, it was verified by WB that 11β-HSD1 significantly enhanced the expression of YAP *in vitro*, while verteporfin, a YAP inhibitor, successfully suppressed YAP expression, which reversed osteoclast differentiation even with increased 11β-HSD1 expression (Figure 6F). Finally, TRAP staining revealed that verteporfin can successfully inhibit the activation of osteoclasts (Figure 6G). These data above suggest 11β-HSD1 promotes osteoclastogenesis through regulation of Hippo signaling pathway and YAP.

## Discussion

Postmenopausal osteoporosis, which is characterized by the decrease of bone mineral density in women after menopause, has become a serious public health problem. Effective therapies, such as estrogen replacement therapy, have been sought for this disease since the 1940s^1^. However, estrogen cannot be used as a long-term treatment for osteoporosis due to the side effects. Since the development of denosumab, new drugs for osteoporosis treatment have been discovered by careful analysis of rare bone diseases and bone cell biology, particularly subcellular assessment of the osteoclasts^28^.

Human skeleton expresses 11β-HSD1 primarily within the osteoblasts^21^. Elevated 11β-HSD1 in osteoblasts inhibits osteogenic differentiation^18^, ^29^. BVT-2733, as a selective inhibitor of 11β-HSD1, can help osteoblasts against endogenous glucocorticoid induced dysfunction^27^. However, according to a clinical study, selective 11β-HSD1 inhibitors have no significant effect on bone formation markers^30^. At the same time, the use of a nonselective 11β-HSD1 inhibitor (carbenoxolone) resulted in a reduction in bone resorption markers^21^. We therefore investigated the role of 11β-HSD1 in osteoclasts and we hope it will be a new target for osteoclast inhibition in osteoporosis.

At present, there are many options for the treatment of osteoporosis, but some of them have certain side effects after long-term use. For example, long-term bisphosphonate administration can even inhibit bone formation and result in nonunion after fracture^31^. At the same time, many studies have shown that a variety of anti-osteoporosis drugs can be used sequentially or in combination for better therapeutic effects, so we are very hopeful to obtain more diverse anabolic pathways in treatment^32^. Therefore, there are many articles trying to find more accurate and specific targets for the treatment of osteoporosis, such as suitable microRNA as targets^33^. However, such protocols are difficult to validate and translate *in vivo*.

Bone remodeling is tightly regulated by a variety of factors, including hormones, growth factors, cytokines, and signaling transduction pathways^34^. In our study, inhibition of 11β-HSD1 by the small molecule drug BVT-2733 could be used for future transformation. Likewise, most of the current research methods for small molecule drugs are to administer drugs to mice by oral administration or injection^35^, and it is difficult to study the influence of other tissues or cells on the drug. Liu et al^36^ reported that selective inhibition of acetylated histone BRD4 inhibited osteoclathogenesis and ovariectomized osteoporosis. But such protocols cannot study the effects of drugs on a single cell line. In our study, we used CTSK-Cre mice to study the changes of 11β-HSD1 in the mouse bone marrow cavity, which more precisely confirmed the role of 11b-HSD1 in osteoclasts under OVX condition, and better explained the role of 11β-HSD1 in osteoporosis.

In our study, knock out of 11β-HSD1 in osteoclasts successfully rescued bone loss caused by OVX surgery in mice. Firstly, we found that H type vessel, a kind of preosteoclast-induced angiogenesis, was inhibited by 11β-HSD1 through PDGF-BB. Xie et al.^15^ demonstrated that H type vessels are a potential therapeutic target for postmenopausal osteoporosis. Currently, some literatures have reported that age-related loss of H type vessels plays a critical role in the pathogenesis of osteoporosis^37^. It has also been studied some drugs preventing bone loss in OVX mice by promoting preosteoclast PDGF-BB-induced type H vessel formation^38^. These experiments all proved that the osteoclast lineage was the major source of PDGF-BB in bone marrow. Since H type vessels are closely associated with the progression of osteoporosis, it has been suggested by many that H-type vessels may be a treatment option for osteoporosis^39^. For example, Xu et al.^40^ found that the SLIT3 pathway in osteoblasts can regulate the formation of H blood vessels and thus target the treatment of osteoporosis. Aburahman et al.^41^ reported that loading increases the formation of H-type vessels and inhibits bone loss caused by OVX. Our studies have shown that 11β-HSD1 can regulate osteoclast maturation and osteoporosis, so we speculate that 11β-HSD1 and H type vessel may have a certain relationship.We found that 11β-HSD1 inhibited the expression of H type both *in vitro* and *in vivo*, which might be due to the fact that 11β-HSD1 accelerated the maturation of osteoclasts and reduced the expression of PDGF-BB.

Furthermore, we demonstrated that osteoclast differentiation may be mediated by the Hippo pathway. When the Hippo signaling pathway is not activated, YAP and TAZ enter the nucleus and exert biological functions^42^, ^43^. YAP expression is controlled by several well-known osteoclast-related signaling cascades^44^. Zhao et al^45^ found that is essential for RANKL-induced osteoclast differentiation and activity. However, this study only endogenously confirmed the effect of 11β-HSD1 on OVX osteoporosis, the deeper mechanism between Hippo pathway and osteoclast activation has not been explored, and lack of 11β-HSD1 inhibitor drugs and other treatment of osteoporosis, which will be enriched in future studies.

In conclusion, the expression of 11β-HSD1 in osteoclasts can promote osteoclastogenesis through a variety of ways, which may serve as a new target for the treatment of osteoporosis.

## Methods

### Animals

We purchased C57BL/6J mice from Animal Facility of Soochow University (Suzhou, China). The animal handling and surgical procedures were conducted in accordance with protocols approved by the Ethics Committee at the First Affiliated Hospital of Soochow University.

After anesthesia, ovariectomy (OVX) was performed on 2-month-old female mice for the osteoporosis model as previously reported^46^.

11β-HSD1 knock out (KO), knock in (KI) and Floxed 11β-HSD1 (11β-HSD1^fl/fl^) mice were a gift from Prof. Lv Shan (Jiangsu Province Hospital, Nanjing, China). Ctsk-Cre mice were a gift from Prof. Chen Jianquan (Soochow University, Suzhou, China). Male C57BL/6J mice was taken as male KO and KI control, which were sacrificed at 4-month-old. Ctsk-Cre mice were crossed with 11β-HSD1^fl/fl^. We determined the genotype of transgenic mice (Ctsk-Cre:: 11β-HSD1^fl/fl^) by PCR analysis of genomic DNA isolated from mouse tails. Genotyping for the Cre transgene was performed by PCR with the primers as below. The representative PCR images were showed in Figure S1. The female Ctsk-Cre::11β-HSD1^fl/fl^ mice were taken the OVX surgery and 11β-HSD1^fl/fl^ mice were treated as control.

### Micro-CT

Animals in each group were executed at weeks 8, and the femurs of mice were removed and fixed with 4% paraformaldehyde solution. High-resolution micro-CT (SkyScan 1176, SkyScan, Aartselaar, Belgium) bone scans were performed on mouse femurs with the following settings, namely, 65 kV, 385 mA, and 0.5 mm Al filter. Three-dimensional (3D) reconstruction was performed for quantitative comparison and analysis of the following parameters: bone volume/total volume (BV/TV) and trabecular separation/spacing (Tb.Sp) to evaluate the degree of osteoporosis. To be specific, 1/10 of the femur length below the growth plate were measured for 3D reconstruction and trabecular bone quantification, and 20 slices of the cortex bone area in the middle of the femur were reconstructed for the 3D model.

### Histomorphometry

For histological evaluation, after completion of the Micro-CT scan, the femurs were decalcified in 10% EDTA at room temperature for 2 weeks. After decalcification was completed, the femurs were paraffin embedded, followed by sectioning and ready for hematoxylin-eosin (H&E, Solarbio) staining. The degree of osteoporosis was observed and assessed microscopically. To identify osteoclasts, sections were stained for tartrate-resistant acid phosphatase (TRAP, Sigma) as instructions.

### Immunofluorescent staining

The expression of TRAP (Santa Cruz), 11β-HSD1 (Proteintech), Endomucin (Santa Cruz), CD31 (R&D), PDGF-BB (Abcam) and YAP (Abcam) in femoral tissues was analyzed by immunofluorescence. The staining method was similar to that of our previous published article^47^. Finally, the positively stained cells were scanned using fluorescence microscope (Zeiss Axiovert 200; Carl Zeiss Inc., Thornwood, NY, USA). Quantitative analysis was done with the aid of ImageJ (National Institutes of Health, NIH)^48^.

### Immunohistochemical staining

Immunohistochemical staining was carried out as described^49^. Specifically, 11β-HSD1 (Proteintech) and YAP (Cell Signaling Technology) were used as primary antibodies.

### Osteoclast induction test

C57BL/6J BMMs were isolated and cultured as reported^46^. We seeded BMMs at a density of 2×10^4^ in 48-well plates while promoting BMMs apposition with M-CSF (R&D, 30 ng/ml) and stimulated with 50 ng/ml of RANKL (R&D) 24 hours later, and started to observe continuously for the appearance of osteoclasts after the fifth day. BVT-2733 was synthesized by Nanjing Medical University First Affiliated Hospital, Nanjing, China. Verteporfin (Sigma) was used as previous reported^45^.

### Western blot

Equal amounts of protein samples were resolved on a 10% SDS-PAGE gel and transferred onto a nitrocellulose membrane. After blocking with Western Blocking Buffer (Beyotime), the membrane was probed with primary antibodies specific for 11β-HSD1 (Proteintech), YAP (Cell Signaling Technology), CTSK (Proteintech) and GAPDH (Abcam) followed by secondary antibodies (Proteintech), and the signal was detected with chemiluminescence (Cell Signaling Technology) according to the manufacturer's protocols. GAPDH was applied to normalize the protein expression levels.

### Quantitative real-time RT-PCR

Total RNA was prepared using TRIzol (Sigma, T9424) and was reverse transcribed into cDNA with 5X All-In-One RT MasterMix (Abm). RT-qPCR was performed using the iQ SYBR Green Supermix (Bio-Rad, Hercules, CA, USA). The real-time reverse transcriptase RT-PCR reaction was performed with the BioRad CFX96 system. The relative amount of each studied mRNA was normalized to GAPDH levels as a housekeeping gene, and the data were expressed according to the 2-ΔΔCT method. The primer sequences used were listed in Table 3.

### Tube formation

Tubule formation assay for HUVEC was performed as previously described^50^. Briefly, a 24-well plate was polymerized by Matrigel (BD Biosciences, Bedford, MA) for at least 30 min in incubator. HUVECs (2×104) were incubated for 24 h before image taking. The picture were taken under a 100X bright-field microscope.

### Elisa

We determined the concentration of PDGF-BB in the conditioned media by the ELISA Development Kit (Solarbio) according to the manufacturer's instructions.

### Transcriptome analysis

The BMMs were analyzed after activation. The transcriptome sequencing was conducted by OE biotech Co. Ltd. (Shanghai, China). The Gene Ontology enrichment analysis was performed using the OE suite of online tools (https://cloud.oebiotech.cn/task/).

### Statistical analysis

All data was presented as means ± standard deviation. Student's t-test was used to analysis the difference within only two groups and one-way analysis of variance (ANOVA) are chosen when three or more groups were compared. P < 0.05 was considered statistically significant.

## Supplementary Material

Supplementary figures.

## Figures and Tables

**Table 1:** Sequences of primers employed for PCR

Primer Name	Sequence (5'-3')
Ctsk-Cre-F	GATCTCCGGTATTGAAACTCCAGC
Ctsk-Cre-R	GCTAAACATGCTTCATCGTCGG
11β-HSD1-LoxP-F	GGGGTTCCTGTATCTGCTCATG
11β-HSD1-LoxP-R	GGTCATCTTGGTCGTAGGCTTT
KI-tF3	ATGCCCACCAAAGTCATCAGTGTAG
KI-tR3	AGGCGGGCCATTTACCGTAAGTTA
KI-tF2	GGGCAGTCTGGTACTTCCAAGCT
KI-CAG-5tR2	ATATCCCCTTGTTCCCTTTCTGC
KO-FRT-tF3	AATCTGTAACCCTGTATCCTCCAG
KO-LoxP-tR1	CAGAAGGCAAAGGTGGGCAGAT
KO-LoxP-tF2	GGGGTTCCTGTATCTGCTCATG
KO-LoxP-tR2	GGTCATCTTGGTCGTAGGCTTT
Cre KT 119	TGCCACGACCAAGTGACAGCAATG
Cre KT 120	ACCAGAGACGGAAATCCATCGCTC

**Table 2:** Antibodies used in this study

Antibody	Source	Identifier
11β-HSD1 (IF, IHC, WB)	Proteintech	10928-1-AP
TRAP (IF)	Santa Cruz	sc-376875
Ctsk (WB)	Santa Cruz	sc-48353
GAPDH (WB)	Proteintech	10494-1-AP
Endomucin (IF)	Santa Cruz	sc-65495
CD31 conjugated to Alexa Fluor 488 (IF)	R&D Systems	FAB3628G
PDGF-BB (IF)	Abcam	ab23914
YAP (IF, IHC, WB)	CST	14074

**Table 3:** Sequences of primers employed for RT-PCR

Target gene	Forward primer sequence (5'-3')	Reverse primer sequence (5'-3')
11β-HSD1	CAGAAATGCTCCAGGGAAAGAA	GCAGTCAATACCACATGGGC
ACP5	CACTCCCACCCTGAGATTTGT	CCCCAGAGACATGATGAAGTCA
Cathepsin K	CTTCCAATACGTGCAGCAGA	TCTTCAGGGCTTTCTCGTTC
Nfatc-1	GAGACAGACATCCGGAGGAAGA	GTGGGATGTGAACACGGAAGA
GAPDH	TGCACCACCAACTGCTTGC	GGCATGGACTGTAGTCAGAG
Alp	CCAGCAGGTTTCTCTCTTGG	CTGGGAGTCTCATCCTGAGC
Osteocalcin	TTGAACTGTTTGTTTTGGACCC	CCAACAGACACCAGTTGTAAAG
Osterix	CATCCCTATGGCTCGTGGTA	TGGGTTAAGGGGAGCAAAGT
STAT3	CTTGTCGGTTGGAGGTGTGAGG	AGGGTCTGGAGTCTGGGTTGG
