# Supplementary material for: 11β-Hydroxysteroid Dehydrogenase Type 1 Facilitates Osteoporosis by Turning on Osteoclastogenesis through Hippo Signaling
Source: Int J Biol Sci. 2023 Jul 15;19(11):3628–39. doi: 10.7150/ijbs.82933 (PMC10367550; doi:10.7150/ijbs.82933)
Supplement: Supplementary file 1 — Supplementary figures. [file ijbsv19p3628s1.pdf]

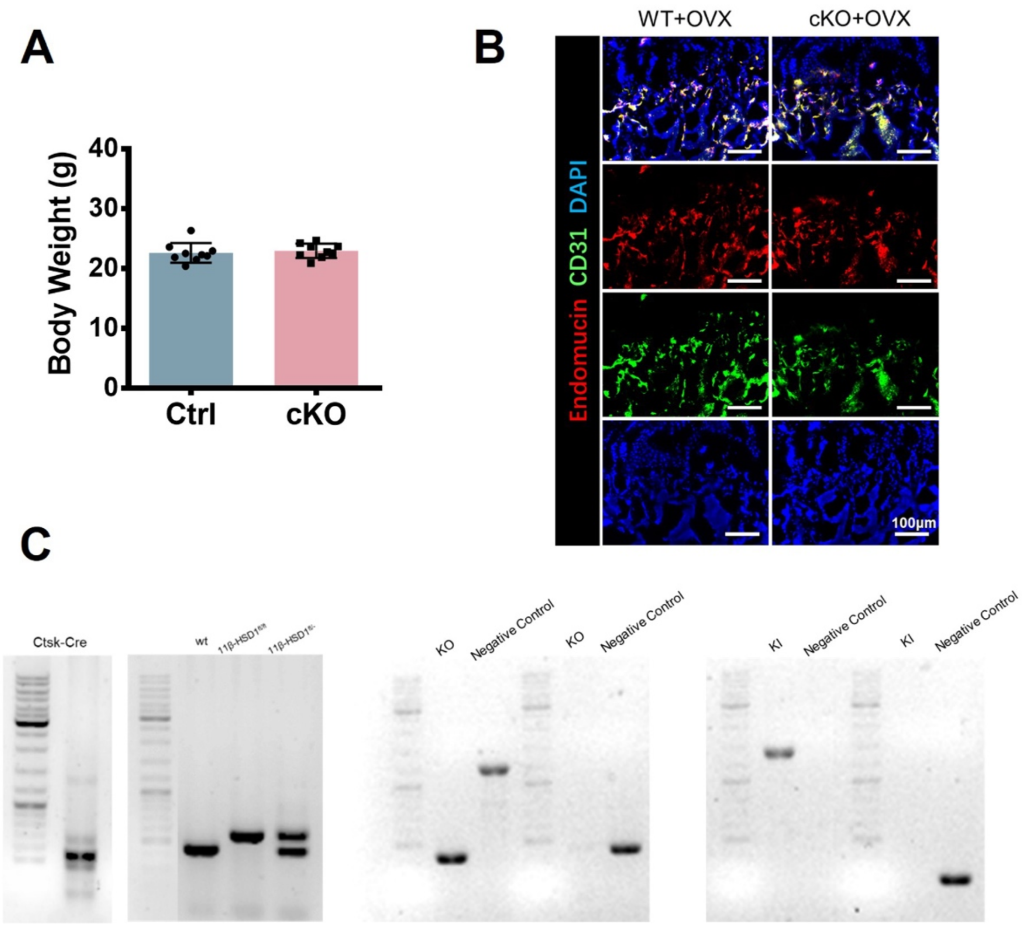

Figure S1

A. Body weight of the control and cKO mice.

B. Representative images of immunostaining of Endomucin (Emcn, red), CD31 (green) and DAPI (blue).

C. Representative images of PCR analysis.

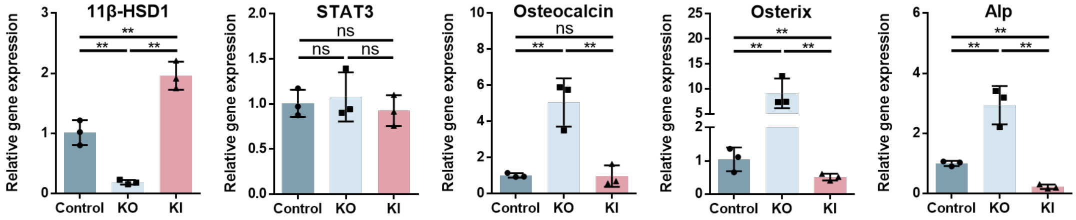

Figure S2 RT-qPCR analysis of 11β-HSD1, STAT3, Osteocalcin Osterix and Alp in BMSCs from wild type, KO and KI groups.
